# Grand Challenges in Eating Behavior Research: Preventing Weight Gain, Facilitating Long-Term Weight Maintenance

**DOI:** 10.3389/fpsyg.2017.00388

**Published:** 2017-03-14

**Authors:** Adrian Meule, Claus Vögele

**Affiliations:** ^1^Department of Psychology, University of SalzburgSalzburg, Austria; ^2^Center for Cognitive Neuroscience, University of SalzburgSalzburg, Austria; ^3^Research Unit INSIDE, Institute for Health and Behavior, University of LuxembourgEsch-sur-Alzette, Luxembourg

**Keywords:** eating behavior, obesity, prevention, intervention, eHealth, nudging, tailored treatments

The Specialty Section *Eating Behavior* covers a wide range of topics related to food, nutrition, and eating both in animals and humans. These include cognitive, emotional, physiological, and behavioral responses to food and food-related cues; individual, social, cultural, and developmental factors affecting eating behavior and food choice; interactions of body image perception and physical activity with eating and appetite; eating disorders, and many more. Contributions in all of these areas are welcome.

An area, which embraces most of these topics, concerns one of the biggest challenges of our time: preventing and treating obesity. Obesity is defined as a condition of excessive fat accumulation in adipose tissue to the extent that health may be impaired (World Health Organization, 2000). Although the health threats of obesity have already been described for centuries (Haslam, 2011), obesity has not been a major public health problem until prevalence rates started to rise so extensively over the last decades that it can now be considered a global pandemic (Ng et al., 2014). Obesity is the result of a positive energy balance, that is, energy intake exceeds energy expenditure. While increasing energy expenditure obviously requires engaging in an effortful activity (e.g., exercising), decreasing energy intake appears to be a simpler method at first glance: individuals merely have to eat less food or substitute previously consumed energy-dense foods with other foods that are less energy dense.

While behavioral treatments that aim at increasing physical activity and/or reducing energy intake are successful in promoting behavior change and, therefore, weight loss, the majority of individuals usually regain weight after termination of such programs, often even exceeding the initial weight after a few months or years (Lowe, 2015). The most successful (and long-lasting) non-behavioral weight-loss treatment appears to be bariatric surgery (e.g., gastric bypass; Colquitt et al., 2014). This approach, however, is unsuitable as a panacea for all obese individuals due to substantial downsides such as surgery costs, complications during or after surgery, adverse effects (e.g., dumping syndrome), the need for life-long nutritional supplementation, or overstretched skin as a result of the rapid weight loss (de Zwaan et al., 2014). Thus, there is an urgent need for developing new or modifying existing weight-loss treatments.

Indeed, research on new ways how to decrease unhealthy food intake seems to be flourishing. The recent rise in eHealth technology (e.g., mobile health applications using smartphones or other portable devices, Internet-based interventions) has created an opportunity to implement preventions and interventions to large parts of the population at little cost. It is not surprising, therefore, that recent research is increasingly aiming at integrating eHealth technologies in behavior modification approaches. For example, a growing number of studies examines the use of computerized tasks, such as motor response inhibition/facilitation trainings, approach-avoidance trainings, or attentional bias modification in order to modify dietary choices. These techniques are proposed to change eating behavior by changing motor responses (e.g., approach-avoidance tendencies) toward food, by altering the motivational value of food, by modifying attention to food, and/or by rule-based learning mechanisms (Stice et al., 2016). Other approaches use ecological momentary interventions (e.g., text-messaging and other smartphone-based applications) to incorporate psychosocial treatments in daily routine (Heron and Smyth, 2010; Boh et al., 2016).

EHealth technologies constitute a strategic ally to sustainable development goals and to attaining universal health coverage through enabling equitable access to high quality, safe, effective, and affordable health care services (Hussein, 2015). Nevertheless, just as in traditional behavioral weight-loss treatments, there is still one major obstacle: they require self-regulation. Performing training sessions or using smartphone apps is an effortful process that requires constant motivation and available cognitive resources. In our obesogenic environment, which promotes physical inactivity and provides easy access to cheap, palatable, energy-dense foods, it appears that any kind of weight-loss treatment, which relies on self-regulation, is bound to fail. In his book *Slim by Design*, Brian Wansink even suggests that the solution for mindless eating is not mindful eating because this will not be successful in the long run. Instead, individuals would just need to change their food environment, which leads them to eat too much in the first place, often without them being aware of it (Wansink, 2014).

In line with this view, researchers have long called for population-based prevention approaches that alter exposure and access to high-calorie foods and beverages (Brownell and Frieden, 2009; Gearhardt et al., 2012). For example, increased taxation and limitation of advertising of alcohol and tobacco reduces their consumption, while educational campaigns are largely ineffective (Perry and Creamer, 2014). While substituting caloric soft drinks with non-caloric, sweetened soft drinks has been found to facilitate weight loss (Rogers et al., 2016), extensive food marketing to children (e.g., via advertising on television) is known to increase preference for and consumption of high-calorie foods (Boyland and Halford, 2013). As a consequence, taxation of sugar-sweetened beverages has already been enacted in some countries (Levell et al., 2016; Scarborough et al., 2016), and limiting food advertising, particularly to children, has been demanded as well (Harris et al., 2013). Thus, it is our contention that one of the biggest challenges in the field of eating behavior research is to conceive of and implement both prevention and intervention approaches for obesity that only require minimal effort by the individual and instead “nudge” them to more healthy food choices and reduced energy intake (Thaler and Sunstein, 2008). While more effortful approaches may lead to weight loss, they will likely not lead to long-term weight maintenance. A word of caution concerning the nudge approach: despite its popularity, as evident from the establishment of nudge units at public policy level in a number of countries (e.g., UK, US, Denmark, Singapore, Canada), the systematic investigation and implementation of nudges to create behavior change is still in its infancy and lags behind the somewhat pre-scientific and, therefore, premature enthusiasm by some policy makers.

Another challenge in obesity prevention and intervention is that obese individuals represent a heterogeneous population. For example, Stunkard has described different eating patterns among obese persons as early as 1959, when he differentiated between *eating without satiation, binge eating*, and *night eating* (Stunkard, 1959). These and other eating patterns have since been well-documented in children and adults in experimental studies (e.g., Tanofsky-Kraff et al., 2009; Dalton et al., 2013; Dalton and Finlayson, 2014; Hofmann et al., 2016). In contrast to these findings, however, the majority of studies in both basic and applied obesity research compare obese versus normal-weight individuals, although these groups are merely defined by their body mass index, without regard to their eating behavior. Therefore, it appears inappropriate to consider obese individuals as a homogeneous group, for which all treatments will be equally effective (Carter and Jansen, 2012). Accordingly, a recent study showed that a tailored family-based obesity intervention turned out to be more successful for reducing weight and increasing healthy food intake in children than a usual care program (Taylor et al., 2015). Similarly, it has been suggested that for prevention approaches to be effective, food-policy actions should be tailored to the characteristics of the people they seek to support (Hawkes et al., 2015).

In conclusion, there is much to be gained from the adoption of eHealth technologies for the improvement of existing and development of new prevention and treatment programs, and the recognition of the importance of tailoring programs to the needs of the target population. Nevertheless, much needs to be done in terms of the systematic investigation of nudges, that is, non-fiscal and non-regulatory interventions that steer (nudge) people in a specific direction while preserving choice. Less attention has been paid to “boosts” (Grüne-Yanoff and Hertwig, 2016) conceived as a distinct form of intervention, the ultimate goal of which is to make it easier for people to exercise their own agency in making choices. In any case, for behavior modifications to be successful and durable, it will be crucial to address self-efficacy, a malleable resource, which is of prime importance in the area of health behavior change (Schwarzer, 2008). Self-efficacy is positively associated with persistency, decisiveness, and constructive ways of dealing with failures (i.e., resilience; Bandura, 2004). Hence, through the association with better coping styles and promotion of agency, higher levels of self-efficacy are likely to reduce the negative effects of, for example, health-damaging choice-behaviors on diet and physical activity, above and beyond effects of nudges and boosts.

## Author contributions

All authors listed have made substantial, direct and intellectual contribution to the work, and approved it for publication.

### Conflict of interest statement

The authors declare that the research was conducted in the absence of any commercial or financial relationships that could be construed as a potential conflict of interest.

## References

[B1] Bandura A. (2004). Health promotion by social cognitive means. Health Educ. Behav. 31, 143–164. 10.1177/1090198104263660 15090118

[B2] Boh B. Lemmens L. H. J. M. Jansen A. Nederkoorn C. Kerkhofs V. Spanakis G.. (2016). An Ecological Momentary Intervention for weight loss and healthy eating via smartphone and Internet: study protocol for a randomised controlled trial. Trials 17, 1–12. 10.1186/s13063-016-1280-x 27000058 PMC4802730

[B3] Boyland E. J. Halford J. C. (2013). Television advertising and branding. Effects on eating behaviour and food preferences in children. Appetite 62, 236–241. 10.1016/j.appet.2012.01.032 22421053

[B4] Brownell K. D. Frieden T. R. (2009). Ounces of prevention - The public policy case for taxes on sugared beverages. N. Engl. J. Med., 360, 1805–1808. 10.1056/NEJMp0902392 19357400

[B5] Carter F. A. Jansen A. (2012). Improving psychological treatment for obesity. Which eating behaviours should we target? Appetite 58, 1063–1069. 10.1016/j.appet.2012.01.016 22306789

[B6] Colquitt J. L. Pickett K. Loveman E. Frampton G. K. (2014). Surgery for weight loss in adults. Cochrane Database Syst. Rev. 8:CD003641 10.1002/14651858.CD003641.pub4 PMC9028049 25105982

[B7] Dalton M. Finlayson G. (2014). Psychobiological examination of liking and wanting for fat and sweet taste in trait binge eating females. Physiol. Behav. 136, 128–134. 10.1016/j.physbeh.2014.03.019 24662699

[B8] Dalton M. Finlayson G. Esdaile E. King N. (2013). Appetite, satiety, and food reward in obese individuals: a behavioral phenotype approach. Curr. Nutr. Rep. 2, 207–215. 10.1007/s13668-013-0060-4

[B9] de Zwaan M. Georgiadou E. Stroh C. E. Teufel M. Köhler H. Tengler M.. (2014). Body image and quality of life in patients with and without body contouring surgery following bariatric surgery: a comparison of pre-and post-surgery groups. Front. Psychol. 5:1310. 10.3389/fpsyg.2014.01310 25477839 PMC4235262

[B10] Gearhardt A. N. Bragg M. A. Pearl R. L. Schvey N. A. Roberto C. A. Brownell K. D. (2012). Obesity and public policy. Annu. Rev. Clin. Psychol. 8, 405–430. 10.1146/annurev-clinpsy-032511-143129 22224839

[B11] Grüne-Yanoff T. Hertwig R. (2016). Nudge versus boost: how coherent are policy and theory? Minds Mach. 26, 149-183. 10.1007/s11023-015-9367-9

[B12] Harris J. L. Sarda V. Schwartz M. B. Brownell K. D. (2013). Redefining “child-directed advertising” to reduce unhealthy television food advertising. Am. J. Prev. Med. 44, 358–364. 10.1016/j.amepre.2012.11.039 23498101

[B13] Haslam D. (2011). The history of obesity. Clin. Obes. 1, 189–197. 10.1111/j.1758-8111.2011.00031.x 25585909

[B14] Hawkes C. Smith T. G. Jewell J. Wardle J. Hammond R. A. Friel S.. (2015). Smart food policies for obesity prevention. Lancet 385, 2410–2421. 10.1016/S0140-6736(14)61745-1 25703109

[B15] Heron K. E. Smyth J. M. (2010). Ecological momentary interventions: incorporating mobile technology into psychosocial and health behaviour treatments. Br. J. Health Psychol. 15, 1–39. 10.1348/135910709X466063 19646331 PMC2800172

[B16] Hofmann J. Meule A. Reichenberger J. Weghuber D. Ardelt-Gattinger E. Blechert J. (2016). Crave, like, eat: determinants of food intake in a sample of children and adolescents with a wide range in body mass. Front. Psychol. 7:1389. 10.3389/fpsyg.2016.01389 27708598 PMC5030249

[B17] Hussein R. (2015). A review of realizing the Universal Health Coverage (UHC) goals by 2030: Part 2- what is the role of ehealth and technology? J. Med. Syst. 39, 1–10. 10.1007/s10916-015-0255-x 26044851

[B18] Levell P. O'Connell M. Smith K. (2016). Sugary drinks tax: response from the Institute for Fiscal Studies. Lancet 387, 1907–1908. 10.1016/S0140-6736(16)30421-4 27203654

[B19] Lowe M. R. (2015). Dieting: proxy or cause of future weight gain? Obes. Rev. 16, 19–24. 10.1111/obr.12252 25614200

[B20] Ng M. Fleming T. Robinson M. Thomson B. Graetz N. Margono C.. (2014). Global, regional, and national prevalence of overweight and obesity in children and adults during 1980-2013: a systematic analysis for the Global Burden of Disease Study 2013. Lancet 384, 766–781. 10.1016/S0140-6736(14)60460-8 24880830 PMC4624264

[B21] Perry C. L. Creamer M. R. (2014). The childhood obesity epidemic: lessons learned from tobacco. J. Pediatr. 164, 178–185. 10.1016/j.jpeds.2013.07.036 24011763

[B22] Rogers P. Hogenkamp P. De Graaf C. Higgs S. Lluch A. Ness A.. (2016). Does low-energy sweetener consumption affect energy intake and body weight? A systematic review, including meta-analyses, of the evidence from human and animal studies. Int. J. Obes. 40, 381–394. 10.1038/ijo.2015.177 26365102 PMC4786736

[B23] Scarborough P. Briggs A. Mytton O. Rayner M. (2016). The Institute of Fiscal Studies' verdict on a sugary drink tax. Lancet 387:1162. 10.1016/S0140-6736(16)00680-2 26970722

[B24] Schwarzer R. (2008). Modeling health behavior change: how to predict and modify the adoption and maintenance of health behaviors. Applied Psychology, 57, 1–29. 10.1111/j.1464-0597.2007.00325.x

[B25] Stice E. Lawrence N. S. Kemps E. Veling H. (2016). Training motor responses to food: a novel treatment for obesity targeting implicit processes. Clin. Psychol. Rev. 49, 16–27. 10.1016/j.cpr.2016.06.005 27498406

[B26] Stunkard A. J. (1959). Eating patterns and obesity. Psychiatr. Q. 33, 284–295. 10.1007/BF01575455 13835451

[B27] Tanofsky-Kraff M. McDuffie J. R. Yanovski S. Z. Kozlosky M. Schvey N. A. Shomaker L. B.. (2009). Laboratory assessment of the food intake of children and adolescents with loss of control eating. Am. J. Clin. Nutr. 89, 738–745. 10.3945/ajcn.2008.26886 19144730 PMC2646816

[B28] Taylor R. W. Cox A. Knight L. Brown D. A. Meredith-Jones K. Haszard J. J.. (2015). A tailored family-based obesity intervention: a randomized trial. Pediatrics 136, 281–289. 10.1542/peds.2015-0595 26195541

[B29] Thaler R. H. Sunstein C. R. (2008). Nudge: Improving Decisions about Health, Wealth, and Happiness. New Haven, CT: Yale University Press.

[B30] Wansink B. (2014). Slim by Design: Mindless Eating Solutions for Everyday Life. New York, NY: William Morrow and Company.

[B31] World Health Organization (2000). Obesity: Preventing and Managing the Global Epidemic. Geneva: World Health Organization. 11234459

